# Changes in diet, cardiovascular risk factors and modelled cardiovascular risk following diagnosis of diabetes: 1-year results from the ADDITION-Cambridge trial cohort

**DOI:** 10.1111/dme.12316

**Published:** 2013-10-21

**Authors:** L A Savory, S J Griffin, K M Williams, A T Prevost, A-L Kinmonth, N J Wareham, R K Simmons

**Affiliations:** 1 MRC Epidemiology Unit, Cambridge Institute of Public HealthCambridge, UK; 2 East of England Multi-Professional Deanery, Cambridge Institute of Public HealthCambridge, UK; 3 The Primary Care Unit, Cambridge Institute of Public HealthCambridge, UK; 4 King’s College London, Department of Primary Care and Public Health SciencesLondon, UK

## Abstract

**Aims:**

To describe change in self-reported diet and plasma vitamin C, and to examine associations between change in diet and cardiovascular disease risk factors and modelled 10-year cardiovascular disease risk in the year following diagnosis of Type 2 diabetes.

**Methods:**

Eight hundred and sixty-seven individuals with screen-detected diabetes underwent assessment of self-reported diet, plasma vitamin C, cardiovascular disease risk factors and modelled cardiovascular disease risk at baseline and 1 year (*n* = 736) in the ADDITION-Cambridge trial. Multivariable linear regression was used to quantify the association between change in diet and cardiovascular disease risk at 1 year, adjusting for change in physical activity and cardio-protective medication.

**Results:**

Participants reported significant reductions in energy, fat and sodium intake, and increases in fruit, vegetable and fibre intake over 1 year. The reduction in energy was equivalent to an average-sized chocolate bar; the increase in fruit was equal to one plum per day. There was a small increase in plasma vitamin C levels. Increases in fruit intake and plasma vitamin C were associated with small reductions in anthropometric and metabolic risk factors. Increased vegetable intake was associated with an increase in BMI and waist circumference. Reductions in fat, energy and sodium intake were associated with reduction in HbA_1c_, waist circumference and total cholesterol/modelled cardiovascular disease risk, respectively.

**Conclusions:**

Improvements in dietary behaviour in this screen-detected population were associated with small reductions in cardiovascular disease risk, independently of change in cardio-protective medication and physical activity. Dietary change may have a role to play in the reduction of cardiovascular disease risk following diagnosis of diabetes.

## Introduction

Type 2 diabetes is a growing public health problem, associated with a substantial burden of morbidity and mortality 1. Patients with diabetes are two to four times more likely to die from cardiovascular disease than those without the disease 2. Lifestyle behaviours, including diet, are strongly associated with risk of incident diabetes and other cardiovascular disease risk factors. Dietary modification for weight management and for controlling blood glucose, blood pressure and blood lipid levels, is an important first-line treatment option for newly diagnosed patients. Such individuals will also be given physical activity advice and may be considered for pharmacotherapy. Evidence-informed nutritional guidelines for the management of diabetes from Diabetes UK 3 and the American Diabetes Association 4 focus on the reduction of total energy intake, percentage of energy from saturated fat, and sodium intake, alongside increases in fibre, and fruit and vegetable intake. Randomized trials of lifestyle interventions including dietary modification have demonstrated reduced incidence of diabetes in high-risk individuals 5 and improved cardiovascular disease risk factors in those with established diabetes 6,7. Although less well established, research also suggests that dietary changes in individuals with newly or recently diagnosed diabetes may be valuable 8,9. However, the lack of measurement of diet makes it difficult to quantify the contribution that dietary change made to the beneficial effects observed in these trials.

**What’s new?**

- Dietary modification is an important first-line treatment option for clinically diagnosed patients.
- Dietary changes in individuals with screen-detected diabetes may also be valuable. However, the lack of measurement of diet in previous trials makes it difficult to quantify the contribution that dietary change can make to cardiovascular risk reduction in this patient group.
- Improvements in self-reported dietary behaviour and plasma vitamin C over 1 year in our screen-detected population were associated with small reductions in cardiovascular disease risk factors and modelled cardiovascular disease risk, independently of cardio-protective medication and physical activity.
- Dietary change may have a role to play in the reduction of cardiovascular disease risk following diagnosis of diabetes.

Population screening for diabetes has been recommended by several national organizations and the National Health Service (NHS) includes assessment of diabetes in its Health Checks programme 10. Consequently, more individuals will be found earlier in the disease trajectory, where there is little current evidence for treatment recommendations. Furthering our understanding of dietary change and potential cardiovascular disease risk reduction in individuals with screen-detected Type 2 diabetes should inform provision of diabetes care and improve targeting of resources.

ADDITION-Cambridge is a primary care-based study of screening for Type 2 diabetes, followed by a pragmatic open-label cluster randomized controlled trial comparing intensive multifactorial treatment with routine care in patients with screen-detected diabetes. As dietary behaviour was measured by both self-report and with plasma vitamin C, this cohort offers the opportunity to quantify the independent effect of diet on cardiovascular disease risk early in the disease trajectory. We aimed (1) to describe changes in self-reported diet and plasma vitamin C over 1 year and (2) to explore whether change in diet was associated with a reduction in cardiovascular disease risk factors and modelled cardiovascular disease risk in this screen-detected population.

## Patients and methods

The design and rationale for ADDITION-Cambridge have previously been reported 11. In brief, 49 general practice surgeries in the Eastern region of England (26 in the intensive treatment group and 23 in the routine care group) recruited patients through a stepwise screening programme. Individuals were eligible to be invited for screening if they were registered with one of the participating general practices, were aged 40–69 years, not known to have diabetes and with a diabetes risk score of > 0.17 (corresponding to the top 25% of the population distribution 12). Exclusion criteria included pregnancy, lactation, and illness with a life expectancy of less than 12 months or a psychiatric disorder that might invalidate informed consent. In total, 33 539 eligible participants were invited to take part in the screening programme 13. World Health Organization criteria were used to diagnose diabetes 14. Patients with newly diagnosed Type 2 diabetes in the screening phase were eligible to participate in the treatment study, unless their general practitioner indicated that they had contraindications to proposed study medication. Eight hundred and sixty-seven individuals agreed to participate and all respondents provided written informed consent. Ethics approval was granted by the Eastern Multi-Regional Ethics Committee (reference 02/5/54).

Participants detected with Type 2 diabetes were managed according to the treatment regimen to which their practice was allocated: intensive treatment or routine care. In intensive treatment practices, the intensification of diabetes management was achieved through the addition of a number of features to existing diabetes care 11,15. This included funding to support increased frequency of contact between patients and practitioners, dietician referrals, a minimum of three practice-based meetings incorporating case-based academic detailing, target setting, audit and feedback. Also included were treatment algorithms specifying a stepwise target-led drug treatment regime to reduce hyperglycaemia, blood pressure, hyperlipidaemia and microalbuminuria. The intensive treatment programme also included lifestyle advice concerning diet, physical activity and tobacco consumption, and provision of theory-based education materials and glucometers for patients, with training in their use. Routine care practices followed current UK national guidelines for diabetes management 16–18.

### Measurement and outcomes

Baseline and 1-year health assessments included physiological and anthropometric measurements, venesection and the completion of questionnaires. Anthropometric and clinical measurements were undertaken by trained staff following standard operating procedures. These data collection methods have been described previously 11. Systolic blood pressure was calculated as the mean of three measurements after at least 10 min rest, using an automatic sphygmomanometer (Omron M4; Omron, Milton Keynes, UK). Total cholesterol and HDL cholesterol were measured by means of enzymatic techniques (dimension analyser; Dade Behring, Newark, DE, USA). HbA_1c_ was analysed in venous samples by ion-exchange high-performance liquid chromatography on a Tosoh machines (Tosoh Bioscience, Redditch, UK). Modelled 10-year risk of cardiovascular disease was calculated using the UK Prospective Diabetes Study (UKPDS) engine version 3.0 19. Participants with complete data for risk score variables (sex, ethnicity, smoking status, presence or absence of atrial fibrillation, systolic blood pressure, HbA_1c_, total cholesterol, HDL cholesterol) and without a self-reported history of macrovascular disease were assessed.

Standardized questionnaires were used to collect information on socio-demographic characteristics (education, socio-economic status) and lifestyle habits (smoking status, alcohol consumption). Data on dietary behaviour was collected using a validated food frequency questionnaire 20. This self-report questionnaire is designed to measure usual food intake during the past year and asks for the average intake of specific foods. Data on total energy, fat, fibre, salt and fruit and vegetable intake were extracted from the questionnaire. Dietary behaviour was also measured using plasma vitamin C, a previously validated biomarker for fruit and vegetable intake, reflecting recent dietary intake of vitamin C 20. Levels were established using a Fluoroskan Ascent FL fluorometer (Thermo Scientific, Wilmington, DE, USA). The validated European Prospective Investigation of Cancer (EPIC)-Norfolk physical activity questionnaire 21 was used to collect self-report data on total physical activity.

### Statistical analyses

Baseline and follow-up characteristics were summarized using means and medians. We compared groups between baseline and follow-up using McNemar’s test for categorical data, paired *t*-tests for continuous data and Wilcoxon signed rank test for non-normally distributed continuous variables. Diet change was calculated by subtracting baseline from follow-up values for each of the dietary outcomes (self-reported fruit, vegetable, energy and fat intake, and plasma vitamin C). For fruit and vegetable intake, the unit of measurement was expressed as 80 g/day, which is roughly equivalent to one portion of fruit or vegetables to allow ease of interpretation in regression models. Similarly, the unit for energy was expressed as 100 kcal/day. Multivariable linear regression models were used to describe the association between change in diet and cardiovascular disease risk factors and modelled cardiovascular disease risk at 1 year; results are reported as unstandardized β-coefficients. The residuals of all linear regression models were checked to ensure that they were consistent with a normal distribution. All models adjusted for baseline dietary behaviour, age, sex, randomization group, socio-economic status, change in smoking status, change in self-reported total physical activity levels, change in alcohol consumption, and change in medication where applicable. For analyses in which total cholesterol or HDL cholesterol was the outcome, the model was adjusted for change in lipid-lowering medication from baseline to follow-up; similarly, for systolic blood pressure, models were adjusted for change in anti-hypertensive medication and, for HbA_1c_, for prescription of glucose-lowering medication at 1 year. In the analysis of 10-year modelled cardiovascular risk, participants with a prior cardiovascular disease event were excluded (*n* = 86) and models included adjustment for change in lipid-lowering, anti-hypertensive and glucose-lowering medication. Where the exposure was self-reported dietary fat intake (per cent of total energy intake from dietary fat), total energy intake was included as a covariate to enable effects on change in cardiovascular disease risk factors to be estimated per 1% increase in fat 22. This nutrient density approach is useful because it represents dietary public health recommendations, which are expressed in terms of percentage of energy from fat. Models were also run separately by trial arm. As results were largely similar, the data were pooled and results from linear regression models based on data from the whole cohort are presented. Type I error was set at 0.05. All data were analysed using Stata version 11 (StataCorp., College Station, TX, USA).

## Results

Baseline characteristics of ADDITION-Cambridge participants with complete data at baseline and follow-up (*n* = 736) are presented in Table 1. Participants who did not attend follow-up were more likely to come from a manual socio-economic class and were more likely to smoke than those who did attend. Non-attenders also reported lower levels of fruit intake and vegetable intake compared with attenders. For all other baseline characteristics, there were no significant differences between attenders and non-attenders (data not shown).

**Table 1:** Characteristics of ADDITION-Cambridge participants with complete data at baseline and 1-year follow-up (*n* = 736)

Characteristics	Baseline	Missing data	One-year follow-up	Missing data
Socio-demographic				
Age, years	61.1 (7.1)	0/736	–	–
Caucasian ethnicity, *n* (%)	712 (96.7)	0/736	–	–
Occupation, *n* (%)		16/736		
Managerial and professional	248 (34.4)		–	–
Intermediate	165 (22.9)		–	–
Routine and manual	307 (42.6)			
Health behaviours				
Current smoker, *n* (%)	125 (17.0)	0/736	108 (14.9)*	12/736
Median (interquartile range) alcohol intake, units/week	3 (0–10)	11/736	3 (0–9)*	19/736
Self-reported total physical activity, metabolic equivalent h/day	29.4 (9.8)	0/736	29.5 (10.0)	11/736
Prescribed medication				
Lipid-lowering medication, *n* (%)	172 (23.4)	0/736	404 (55.4)*	7/736
Anti-hypertensive medication, *n* (%)	408 (55.4)	0/736	485 (66.5)*	7/736
Glucose-lowering medication, *n* (%)	2 (0.3)	0/736	220 (30.2)*	7/736
Clinical variables				
BMI, kg/m^2^	33.4 (5.6)	4/736	32.3 (5.6)*	3/736
Waist circumference, cm				
Men	114.2 (12.9)	0/453	111.4 (12.9)*	2/453
Women	107.5 (13.0)	1/283	103.9 (13.0)*	1/283
Systolic blood pressure, mmHg	141.7 (19.9)	2/736	136.3 (18.5)*	4/736
Total cholesterol, mmol/l	5.4 (1.1)	16/736	4.5 (1.0)*	3/736
HDL cholesterol, mmol/l	1.19 (0.33)	16/736	1.22 (0.34)*	3/736
HbA_1c,_ mmol/mol	56	18/736	48	10/736
HbA_1c,%_	7.3 (1.7)	18/736	6.5 (0.9)*	10/736
Modelled 10-year cardiovascular disease risk,%†	30.9 (14.5)	23/650	25.5 (12.7)*	35/650
Plasma vitamin C, μmol/l	52.7 (22.3)	75/736	54.4 (23.9)*	27/736
Self-reported dietary intake				
Energy intake, kcal/day	1943 (684)	4/736	1693 (559)*	17/736
Median (interquartile range) energy intake, kcal/day	1840 (1493–2339)	4/736	1622 (1316–2012)*	17/736
Fruit intake, g/day	252.9 (213.6)	35/736	298.6 (216.9)*	44/736
Median fruit intake, g/day	210.7 (109.1–336.9)	35/736	255.9 (149.7–396.4)*	44/736
Vegetable intake, g/day	211.5 (123.9)	38/736	234.4 (140.7)*	56/736
Median (interquartile range) vegetable intake, g/day	188.1 (127.1–266.2)	38/736	210.0 (145.6–291.2)*	56/736
Fruit and vegetable intake (combined), g/day	461.9 (271.9)	65/736	529.5 (287.8)*	79/736
Median (interquartile range) fruit and vegetable intake (combined), g/day	407.1 (270.9–586.3)	65/736	483.1 (344.8–658.3)*	79/736
Fat,% of total energy intake	32.2 (6.2)	4/736	30.6 (6.2)*	17/736
Median (interquartile range) fat,% of total energy intake	32.4 (28.1–36.4)	4/736	30.7 (26.3–34.9)*	17/736
Englyst fibre [non-starch polysaccharides (NSP)] intake, g/day	16.9 (6.7)	4/736	18.3 (7.4)*	17/736
Median (interquartile range) englyst fibre (NSP) intake, g/day	15.9 (12.2–20.1)	4/736	17.3 (13.4–21.6)*	17/736
Sodium intake, mg/day	2782 (1083)	4/736	2661 (1042)*	14/736
Median (interquartile range) sodium, g/day	2.7 (2.0 to 3.3)	4/736	2.5 (2.0–3.2)*	14/736

All values are means (sd) unless otherwise indicated.

* *P* < 0.05 from McNemar’s test for categorical variables, paired *t*-test for normally distributed continuous variables and Wilcoxon signed rank test for non-normally distributed continuous variables for baseline vs. follow-up (separately in men and women).

† Participants with a prior cardiovascular disease event (*n* = 86) were excluded.

The mean (sd) age of participants was 61.1 (7.1) years. There were 453 (63%) men, 97% were of Caucasian ethnicity and 43% were in routine or manual occupations. At baseline, participants reported consuming a median of three alcohol units/week and 17% were current smokers. A significant proportion of the cohort were prescribed lipid-lowering (23%) and anti-hypertensive medication (55%). On average, the cohort was obese (33.4 kg/m^2^) with an adverse cardiovascular risk profile. The mean 10-year modelled cardiovascular disease risk was 31%. The self-reported mean daily energy intake was 1943 kcal/day and combined daily fruit and vegetable intake was 462 g/day.

### Change in cardiovascular disease risk factors, modelled cardiovascular disease risk and diet (Table 1)

ADDITION-Cambridge participants reduced their waist circumference and BMI from baseline to follow-up. They also reduced their alcohol consumption and a significant proportion gave up smoking. Reductions were also seen for total cholesterol, systolic blood pressure, HbA_1c_ and modelled cardiovascular disease risk, alongside increases in the prescription of lipid-lowering, anti-hypertensive and glucose-lowering medication. HDL cholesterol values increased. No significant changes in physical activity levels were reported.

Individuals reported reduced energy, fat and sodium intake at 1 year compared with baseline. There were significant increases in self-reported fruit, vegetable and fibre intake. There was a small but significant increase in plasma vitamin C levels (+ 2.0 μmol/l). Results were unaffected by excluding the 69 participants who reported regularly consuming tablets containing vitamin C.

### Association between change in diet, cardiovascular disease risk factors and modelled cardiovascular disease risk (Table 2)

Increases in self-reported fruit intake were associated with small reductions in waist circumference, HbA_1c_ and total cholesterol, while increased vegetable intake was associated with an increase in BMI and waist circumference. A reduction in fat and energy intake was associated with a reduction in HbA_1c_ and waist circumference, respectively. The largest number of associations was seen for plasma vitamin C, where an increase was associated with a significant reduction in BMI, waist circumference, HbA_1c_ and modelled cardiovascular disease risk. A reduction in sodium was associated with a reduction in total cholesterol and modelled cardiovascular disease risk. There were no associations between change in dietary factors and systolic blood pressure or HDL cholesterol at 1 year. Change in fibre intake from baseline to 1 year was not associated with any cardiovascular disease risk factor outcome.

**Table 2:** Association between change in dietary intake, cardiovascular disease risk factors and modelled cardiovascular disease risk at 1-year follow-up in the ADDITION-Cambridge trial cohort*†

	BMI (kg/m^2^)	Waist circumference (cm)	Systolic blood pressure (mmHg)	HbA_1c_ (mmol/mol)	Total cholesterol (mmol/mol)	HDL cholesterol (mmol/l)	Ten-year modelled cardiovascular disease risk (mmol/mol)
∆ Fruit intake (80 g/day)	–0.132 (–0.302 to 0.037)	–0.414 (–0.816 to –0.012)	0.309 (–0.260 to 0.878)	–0.040 (–0.066 to –0.013)	–0.036 (–0.065 to –0.006)	–0.011 (–0.022 to 0.001)	0.001 (–0.002 to 0.009)
∆ Vegetable intake (80 g/day)	0.620 (0.323 to 0.918)	1.180 (0.456 to 1.905)	–0.470 (–1.547 to 0.608)	0.000 (–0.050 to 0.048)	0.035 (–0.020 to 0.091)	–0.014 (–0.035 to 0.007)	0.002 (–0.003 to 0.008)
∆ Fat intake (% of total energy intake)	0.005 (–0.067 to 0.077)	0.039 (–0.131 to 0.209)	0.004 (–0.245 to 0.253)	0.012 (0.001 to 0.023)	0.013 (–0.001 to 0.026)	–0.001 (–0.006 to 0.004)	0.001 (0.000 to 0.002)
∆ Energy intake (100 kcal/day)	0.078 (–0.008 to 0.164)	0.207 (0.005 to 0.409)	–0.026 (–0.314 to 0.261)	0.010 (–0.004 to 0.023)	0.009 (–0.006 to 0.024)	–0.002 (–0.008 to 0.003)	0.001 (0.000 to 0.003)
∆ Plasma vitamin C (μmol)	–0.028 (–0.048 to –0.007)	–0.102 (–0.151 to –0.054)	–0.046 (–0.118 to 0.026)	–0.004 (–0.007 to 0.000)	–0.003 (–0.007 to 0.001)	0.001 (–0.000 to 0.003)	–0.001 (–0.001 to 0.000)
∆ Englyst fibre [non-starch polysaccharides (NSP)] intake, g/day	0.005 (–0.060 to 0.070)	–0.044 (–0.198 to 0.110)	–0.059 (–0.277 to 0.160)	–0.005 (–0.014 to 0.006)	–0.002 (–0.014 to 0.010)	–0.002 (–0.007 to 0.003)	0.000 (–0.001 to 0.002)
∆ Sodium intake, g/day	0.307 (–0.140 to 0.754)	0.689 (–0.364 to 1.743)	0.190 (–1.318 to 1.700)	0.046 (–0.025 to 0.117)	0.087 (0.008 to 0.166)	–0.013 (–0.044 to 0.017)	0.008 (0.001 to 0.016)

* All models are adjusted for age, sex, trial group, socio-economic status, baseline phenotype, change in total physical activity, change in alcohol intake, change in smoking and change in medication (where relevant).

† Fat intake models also include energy intake as a covariate.

## Discussion

We observed improvements in self-reported dietary behaviour over 12 months in a population of patients newly diagnosed with diabetes in the East of England. These changes were associated with small reductions in cardiovascular disease risk factors and modelled cardiovascular disease risk after allowing for changes in self-reported cardio-protective medication and physical activity. Our results suggest that dietary change may have a role to play in cardiovascular disease risk reduction in individuals with screen-detected diabetes in the first year following diagnosis.

Reductions in cardiovascular disease risk factors have been observed in patients with diabetes enrolled in lifestyle interventions 6–9 and in high-risk individuals enrolled in diabetes prevention programmes 5. In the Diabetes Prevention Programme 23, weight loss was the dominant predictor of reduced risk of progression to diabetes. A lower per cent of dietary calories from fat and increased physical activity independently predicted weight loss. Similarly, weight loss predicted reduced progression to diabetes in the Finnish Diabetes Prevention Study 24. The predictors of a reduction in weight were a low intake of total fat and high dietary fibre intake. More recently, in the Early Activity in Diabetes (Early ACTID) trial, 593 patients recently diagnosed with diabetes were randomized to (1) usual care (control), (2) an intensive diet intervention (6.5 h of individual counselling by a dietician/nurse over 1 year) or (3) an intensive diet intervention plus a pedometer-based activity programme 8. After 12 months, there were significant improvements in glycaemic control, insulin resistance and body weight in both intervention groups compared with the control group; however, the addition of the activity intervention conferred no extra benefit. One-year results from the Diabetes Education and Self Management for Ongoing and Newly Diagnosed (DESMOND) study 9, undertaken in newly diagnosed Type 2 diabetes, suggested that a structured education programme, including a focus on lifestyle factors such as food choices, was associated with reductions in weight and modelled cardiovascular disease risk after adjustment for baseline values and cluster effect. However, as dietary behaviour was not recorded, the independent effects of diet on cardiovascular disease risk factors could not be quantified in the Early ACTID and DESMOND trials.

Significant increases in self-reported fruit and vegetable intake over 12 months were mirrored by a small increase in plasma vitamin C in our cohort. The largest number of statistically significant associations were seen for associations between plasma vitamin C and cardiovascular disease risk factors, rather than for associations with self-reported dietary intake. This may reflect relative precision of measurement and confirms findings from other studies. For example, in the large population-based EPIC-Norfolk cohort, a much stronger inverse association was observed between plasma vitamin C and diabetes risk (odds ratio 0.38, 95% CI 0.28–0.52), than for self-reported fruit and vegetable intake and diabetes risk (odds ratio 0.78, 95% CI 0.60–1.00) 25. The importance of dietary change following diagnosis may have been underestimated in previous studies limited by self-report measures.

### Strengths and limitations

Anthropometric and clinical measurements were undertaken by trained staff following standard operating procedures. Diet was measured across a number of different domains (fruit/vegetable, energy, fat, salt and fibre intake), using a validated food frequency questionnaire. In addition, the study included measurement of plasma vitamin C, which is not endogenously produced and therefore provides a robust measurement of consumption. In order to examine the independent effects of dietary change on cardiovascular disease risk, a number of variables were adjusted for, including change in medication use and change in total physical activity between baseline and 1 year, which might have impacted on cardiovascular disease risk at follow-up. Other intervention studies have not been able to adjust for these factors 8,9,26–28. The study is of larger size and longer duration than many studies in patients with Type 2 diabetes, which are typically limited to less than 1 year. Nearly half of all practices approached agreed to participate 13 and, as general practice registers typically cover 99% of all residents living in England, ADDITION-Cambridge participants were drawn from a large population-based sample, ensuring generalizability to similar settings.

Extrapolation of our results to more deprived and ethically diverse settings may be limited in light of the non-random recruitment of general practices from a single geographical region (Eastern England). Some ‘healthy volunteer’ bias may also be present as non-attenders at 1-year follow up exhibited more unfavourable lifestyle habits than attenders. We conducted multiple significance tests (> 20) of the association between change in diet and cardiovascular disease risk factors, which may have led to an increased risk of type 1 errors. Indeed, we did observe a few associations between self-reported dietary measures and cardiovascular disease risk factors, which were not in the expected direction of effect. A further caution is the use of a self-report food frequency questionnaire, which may have been subject to more error and bias than anthropometric and biochemical measures 29. The food frequency questionnaire used in this study has previously been shown to overestimate fruit intake and the food frequency questionnaire and plasma vitamin C had the weakest correlation compared with other dietary assessment methods 20. Further, as most ADDITION-Cambridge participants were obese, energy intake is likely to have been under-reported in this cohort. This limitation may have been attenuated if the degree of bias was consistent at both time points. Using behaviour change as the exposure of interest would therefore be reliable. Indeed, the food frequency questionnaire is comparable with multiple-day diet records when assessing dietary change 30. However, the degree of misclassification may have been different at baseline and 1-year follow-up. Participants would have received dietary advice following diagnosis and might therefore be more influenced by social desirability bias at follow-up. Despite these challenges, we noted significant improvements in cardiovascular disease risk associated with change in plasma vitamin C, which removes the reliance on self-report data 31, suggesting we did observe some real associations. Future studies might use additional biomarkers for nutritional status to improve the accuracy of dietary measures.

## Conclusion

Significant improvements in dietary behaviour over 1 year were associated with small reductions in cardiovascular disease risk factors and modelled cardiovascular disease risk in a population of patients newly diagnosed with Type 2 diabetes in the East of England. These improvements were independent of change in cardio-protective medication and physical activity. This suggests that dietary change may have a role to play in the reduction of cardiovascular disease risk following diagnosis of diabetes.

### Funding sources

ADDITION-Cambridge was supported by the Wellcome Trust (grant reference no: G061895), the Medical Research Council (grant reference no: G0001164), the National Institute for Health Research (NIHR) Health Technology Assessment Programme (grant reference no: 08/116/300), National Health Service R&D support funding (including the Primary Care Research and Diabetes Research Networks) and the National Institute for Health Research. SJG receives support from the Department of Health NIHR Programme Grant funding scheme (RP-PG-0606-1259). The views expressed in this publication are those of the authors and not necessarily those of the NHS, the NIHR or UK Department of Health. Bio-Rad provided equipment for HbA_1c_ testing during the screening phase.

### Competing interests

None declared.
